# Diagnostic and Therapeutic Challenges of a Large Pleural Inflammatory Myofibroblastic Tumor

**DOI:** 10.1155/2012/102196

**Published:** 2012-12-31

**Authors:** Judith Loeffler-Ragg, Johannes Bodner, Martin Freund, Michael Steurer, Christian Uprimny, Bettina Zelger, Christian M. Kähler

**Affiliations:** ^1^Pneumology/USPH Innsbruck, Department of Internal Medicine VI, Innsbruck Medical University, Anichstraße 35, 6020 Innsbruck, Austria; ^2^Clinic of Visceral, Transplant, and Thoracic Surgery, Innsbruck Medical University, Innsbruck, Austria; ^3^Department of Radiology, Innsbruck Medical University, Innsbruck, Austria; ^4^Department of Internal Medicine V, Innsbruck Medical University, Innsbruck, Austria; ^5^Department of Nuclear Medicine, Innsbruck Medical University, Innsbruck, Austria; ^6^Institute of Pathology, Innsbruck Medical University, Innsbruck, Austria

## Abstract

We report a 48-year-old woman with a pleural pseudoneoplasm requiring different diagnostic and therapeutic strategies. After initial presentation with increasing dyspnoea, temperature, dry cough, and interscapular pain diagnostic processing showed a large mediastinal mass with marked pleural effusion and high metabolic activity in the 18F-FDG-PET/CT. Extensive CT-guided biopsy of the tumor reaching from the visceral pleura into the right upper lobe revealed no malignancy, but a marked inflammatory tissue reaction containing foam cells. Initial empiric antibiotic therapy was temporarily successful. However, in the further course the mass relapsed and was resistant to antibiotics and a corticosteroid trial. With the working hypothesis of an inflammatory myofibroblastic tumor the patient underwent surgical tumor resection, finally confirming the suspected diagnosis. Due to residual disease intravenous immunoglobulins were administered leading to sustained response. This case with a pleural localisation of a large inflammatory pseudotumor with responsiveness to immunomodulation after incomplete resection extends the reported spectrum of thoracopulmonary manifestations of this rare entity.

## 1. Introduction

Inflammatory myofibroblastic tumor (IMT) is a rare non-neoplastic lesion with unknown pathogenesis, comprising less than one percent of all surgically resected lung tumors in adults [1]. They can mimic both clinically and radiologically malignant processes, and a definitive preoperative diagnosis is often difficult to make. These tumors consist of a background proliferation of spindle-shaped mesenchymal cells associated with a variable infiltration with inflammatory cells. IMT most commonly involves the lung and the orbit, but has been reported to occur in nearly every site in the body [2]. Historical synonyms for the disease include inflammatory pseudotumor, plasma cell granuloma, inflammatory myofibrohistiocytic proliferation, histiocytoma, xanthoma, fibroxanthoma, xanthogranuloma, fibrous xanthoma, plasma cell histiocytoma complex, plasmocytoma, and solitary mast cell granuloma [3, 4]. The variety of terms reflects the heterogenous histological patterns that fall under the category of IMT. In this paper we describe the diagnostic and therapeutic approach to a large pleural inflammatory pseudotumor. 

## 2. Case Report

**Figure 1:**
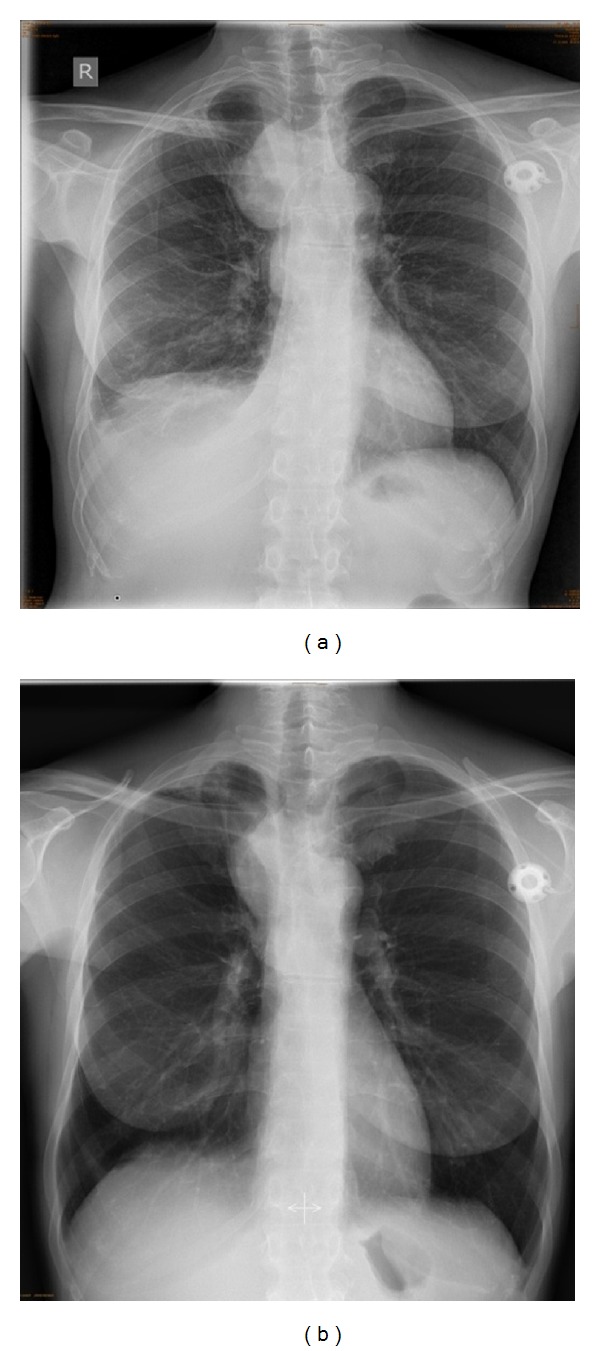
Anteroposterior chest radiograph showing a large homogenous opacity right paramediastinal and right side pleural effusion. (a) Initial presentation. (b) Response to treatment with moxifloxacin four weeks after initial presentation.

**Figure 2:**
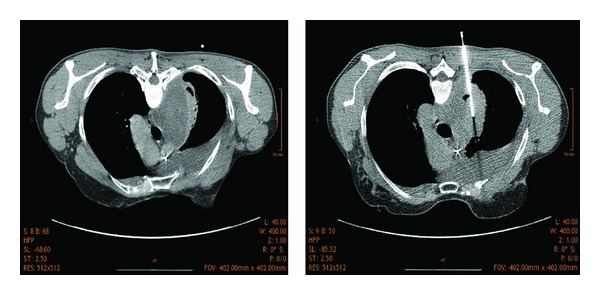
Computed tomography (CT) scan of the chest with CT-guided needle biopsy of a right paramediastinal tumor. In total 10 biopsies were taken from the 8.4 cm large mass.

A 48-year-old woman presented to a peripheral hospital with a 14 days' history of progressive shortness of breath on exertion, dry cough, and interscapular pain. On physical examination the patient displayed reduced breath sounds and a dull percussion note at the right lung base, but was otherwise unremarkable. The initial radiologic work-up revealed a large mediastinal mass measuring 9 cm in size with concomitant marked pleural effusion (Figure 1(a)). The main differential diagnosis was considered to be a malignant disease. Due to a history of breast cancer (invasive ductal carcinoma, ypT1bN1aM0) with following neoadjuvant chemotherapy, surgery and radiation two years before and ongoing adjuvant hormonal therapy with arimidex and zoledronate, the patient was transferred to a gynecological department for further diagnostics. In the following days fever and high CRP levels (up to 27.96 mg/dL; normal range 0.0–0.7 mg/dL) required sequential antibiotic therapy with doxycyclin, piperazillin/tazobactam, and moxifloxacin. Autoimmune parameters (ANA, ANCA) and infectious screening for tuberculosis (T-SPOT), EBV, and toxoplasmosis were negative. Cytology from thoracocentesis revealed no malignant cells. From ten CT-guided needle biopsies of the tumor, which was reaching from the visceral pleura into the right upper lobe (Figure 2), metastasis of breast cancer could be excluded. Because of those indeterminate results the patient was referred to our department. The CT-guided biopsies primarily contained fibrotic and infiltrated parts of pleura and only some parts of normal lung parenchyma. Whereas the intraoperative frozen section was not definitely diagnostic showing an infiltration with small monomorphic cells, the initial H&E histology suggested a macrophage disorder because of monomorphic proliferation of mainly macrophages, some lymphocytes and plasma cells as well as single neutrophiles. No overt signs of malignancy, no nuclear pleomorphism, only rare mitosis, and no necrosis were found. Immunohistochemistry ruled out an underlying neoplastic lesion. The tumorous area was completely negative for epithelial markers namely the pankeratin markers AE3/AE3 and Cam5.2 as well as p63, CK5/6, CK7, and CK20. Calretinin, CD 117, TTF-1, and melanocytic markers as S100, HMB45, and Melan A stained negative too. It showed a prominent macrophage rich, KiM1p and CD 68 positive lesion with single CD4 positive T cells and some CD 79a and CD 138 positive plasma cells. There were no signs of a specific infectious disease such as tuberculosis (microscopy and TBC PCR were negative). H&E morphology and immunophenotype suggested a xanthogranulomatous process and the diagnosis of an inflammatory pseudotumor. Due to the fact that there was only limited material a rebiopsy of the mediastinal mass was recommended, because it was not sure if the material was representative for the whole lesion. The microbiologic workup of the fine needle aspirate was negative for bacteria, mycobacteria, and fungi. 

**Figure 3:**
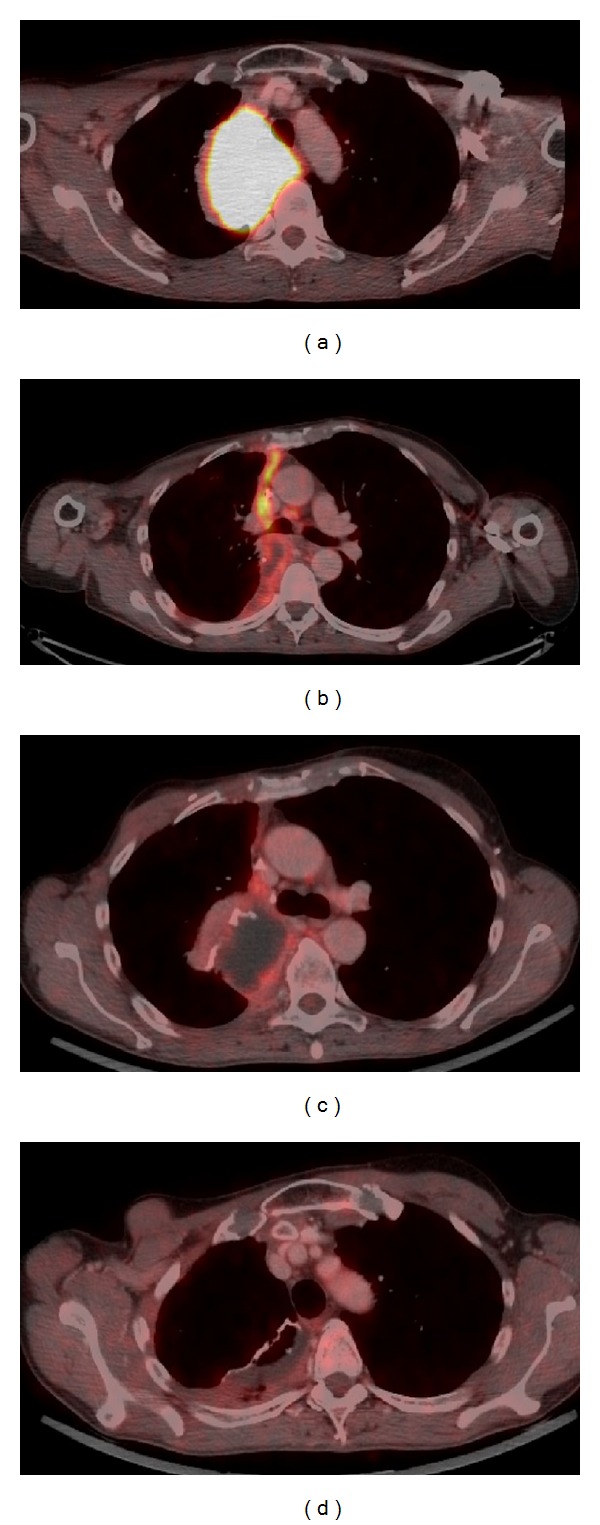
18-FDG PET-CT scan with high FDG uptake in a right paramediastinal mass (a). Postoperative scans showing residual FDG uptake one month after surgery (b), a decrease in intensity after immunomodulation with intravenous immunoglobulins 4 months later (c), and sustained response at one-year followup (d).

**Figure 4:**
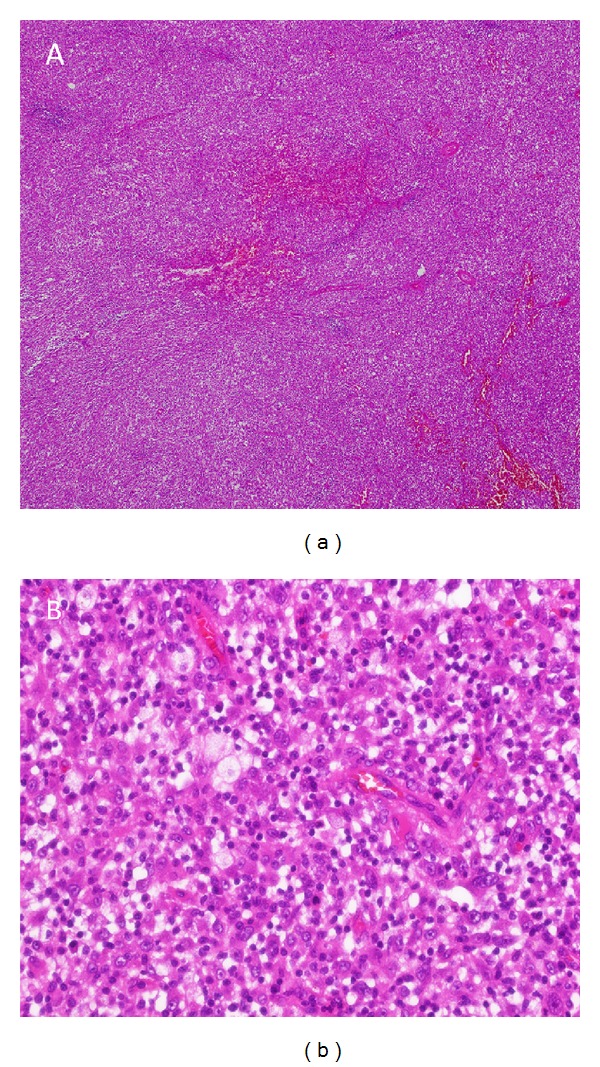
Tumor biopsy showing a dense highly vasculated mixed inflammatory infiltration ((a); H&E, 40x), composed of macrophages with foamy cytoplasm, so called histiocytes, some lymphocytes and plasma cells ((b); H&E, 400x).

Two weeks after the start of moxifloxacin therapy the patient presented herself as afebrile with a marked decrease in CRP (from 27.96 mg/dL to 3.5 mg/dL). Two further weeks later, along with the clinical improvement, a reduction of the size of the mediastinal mass by one-half was noted (from 6.2 × 8.4 cm to 6.2 × 3.4 cm; Figure 1(b)). Because of this clinical course and well documented CT guided needle biopsy routes (excluding sampling error) the working hypothesis of an inflammatory pseudotumor was established. The response to a fourth-generation fluoroquinolone suggested an infectious trigger, which unfortunately could not be proven. Three months later the patient had a clinical worsening with hemoptysis and increase in CRP and tumor size. Therefore, an empirical treatment with methylprednisolone (40 mg/day) was started. 18F-FDG PET-CT scan at that time revealed a very high metabolic activity within the tumor (SUV max.: 41.7) which was suspicious of a malignant disease (Figure 3(a)). Due to a lack of response to corticosteroid therapy within the following 6 weeks, the interdisciplinary board recommended surgical resection of the progressing mass, which advise was followed. The intraoperative situs revealed a yellow, soft, and not well-bordered mass originating from the visceral pleura. The final histology of the pleural tumor and adjacent pulmonary parts from a wedge resection showed a marked inflammatory reaction with foam cells, consistent with the suspected inflammatory myofibroblastic tumor (Figures 4(a) and 4(b)). There was no ALK expression in our case.

The one month postoperative 18F-FDG PET-CT scan revealed in addition to postoperative alterations markedly hypermetabolic lesions (SUVmax.: 20.78) consistent with residual inflammatory disease (Figure 3(b)). Thus, for immunomodulation a single course of intravenous immunoglobulins (IVIG) was administered at a total dose of 1 g/kg divided into two 8-hours infusions within two days. Follow-up18F-FDG PET/CT's showed a good metabolic response with a decreasing metabolic activity after three months (SUVmax.: 9.5) and an almost absent glucose metabolism 12 months after therapy (SUVmax.: 3.2), and apart from that the CRP values remained within the normal range (Figures 3(b) and 3(c)).

## 3. Discussion

In inflammatory pseudotumors the myofibroblast was recognized as the principal cell type and provided the recent classification as inflammatory myofibroblastic tumor [5]. Diverse degrees of inflammatory infiltrates with mixed populations of lymphocytes, plasma cells, histiocytes, and occasional eosinophils define three histological subtypes: organizing pneumonia pattern, fibrous histiocytic, or lymphohistiocytic pattern. In the lung such lesions most commonly present themselves as solitary intrapulmonary nodules, but can also be locally invasive [6]. Pleural manifestation of IMT is rare. There are some cases with pulmonary nodules attached to the pleura, mediastinal lesions with pleural involvement, but to our knowledge such a large pleural lesion has yet not been described [7–10]. It is currently unclear whether IMTs represent a primary inflammatory process versus an underlying low-grade malignancy with a prominent inflammatory response [2, 3]. Theories include a dysregulated cytokine production in response to an infection, because a history of infection is found in a third of patients. Single cases with chronic persistent *Eikenella corrodens* infection, DNA expression of Epstein Barr virus or Human Herpes Virus 8, association with mycobacteria, actinomycetes, nocardiae, and pseudomonas have so far been reported [11–13]. The observation that a chromosomal rearrangement at the site of the anaplastic lymphoma kinase (ALK) gene at band 2p23 is present in about two-thirds of cases supports the hypothesis of a low-grade malignancy with a secondary inflammatory component [5]. Especially case reports with local invasion or metastasis, although very rare, might present such subtypes. In the case presented neither an infection nor ALK expression could be detected. 

Approximately 70% of patients are asymptomatic [14], but like in this case some patients may complain of cough, dyspnea, chest pain, or hemoptysis. As we experienced, radiographic features are not reliable to differentiate IMT from other causes of pulmonary nodules. Thus, diagnosis is often delayed until a diagnostic resection of the indistinct lesion is performed. Because of its propensity to mimic clinically and radiologically a malignant disease, it has been named inflammatory pseudotumor by Umiker WO in 1954 [15]. CT as well as metabolic PET imaging are troubled by false-positive results. It is well known that uptake of radiolabeled glucose is not specific to malignant neoplasms, and may be observed in a variety of tissues with increased glucose consumption. Markedly increased 18F-FDG uptake has already been reported as a feature of IMTs [16, 17]. Also clinical course and the laboratory processing tend to be nonspecific or unremarkable, but the presence of serological evidence of inflammation rather suggests a non-malignant disease. Mildly increased levels of CRP and elevated sedimentation rate have been described in about 50% of cases. The extraordinary high levels in this case may reflect the high “tumor burden” as indicated by metabolic imaging. Despite initial response to antibiotics the disease of our patient progressed. The CRP values were indicative of therapy response as well as relapse. Their value as biomarker for disease monitoring remains uncertain. Complete surgical resection is the treatment of choice for IMT nonresponsive to empirical medicamentous therapy [18]. Most patients can be cured by complete surgical resection, but some lesions become locally invasive and involve the mediastinum, diaphragm, chest wall, vertebral bodies, heart, and major vessels. For patients with progressive disease and unable to have complete surgical resection (e.g., poor surgical candidates, multiple nodules, or unresectable disease), glucocorticoids, radiotherapy, chemotherapy, anti-inflammatory, and immunomodulatory concepts have been used with variable success [7, 19, 20]. In the case presented, due to residual postoperative disease, IVIGs have been administered. This approach was already once shown to be successful in a single patient with resistant inflammatory pseudotumor of the orbit [21]. IVIGs are established modulators of the immune system approved for use in various autoimmune and inflammatory diseases. The precise mechanism by which IVIGs suppress harmful inflammation has yet not been definitively established. Formation of immune complexes, interaction with activating Fc receptors on immune cells such as dendritic cells, T and B cells and monocytes, binding of abnormal host antibodies, activation of the complement system, regulation of macrophage phagocytosis, reduction of levels of tumor necrosis factor-alpha, interleukin 1, 1 beta and 10 are known mechanisms of action [22]. The low profile of adverse events may justify the off-label use of this substance in therapy resistant IMT cases. Two years after therapy our patient is still in remission. In general, long-term follow-up is recommended, as recurrent tumors have been reported as long as 11 years after resection [23]. In conclusion, pleural IPT is a rare condition that should be included in the differential diagnosis of patients with inconclusive inflammatory histology. Because of its rareness, treatment remains empirical, spanning trials from antibiotics, anti-inflammatory or antiproliferative drugs, to surgery and even immunomodulation.
